# Artificial Intelligence Applied to Electrocardiograms Recorded in Sinus Rhythm for Detection and Prediction of Atrial Fibrillation: A Scoping Review

**DOI:** 10.3390/medicina62010199

**Published:** 2026-01-17

**Authors:** Ziga Mrak, Franjo Husam Naji, Dejan Dinevski

**Affiliations:** 1 Department of Cardiology, University Clinical Centre Maribor, Ljubljanska Ulica 5, 2000 Maribor, Slovenia; husamfranjo.naji@ukc-mb.si; 2 Faculty of Medicine, University of Maribor, Taborska Ulica 8, 2000 Maribor, Slovenia; dejan.dinevski@um.si

**Keywords:** atrial fibrillation, ECG, artificial intelligence, deep learning, scoping review

## Abstract

*Background and Objectives:* Subclinical paroxysmal atrial fibrillation (AF) is often undetected by conventional screening strategies, until complications emerge. Artificial intelligence (AI) applied to sinus rhythm electrocardiograms has emerged as a promising tool to identify individuals with occult AF and to predict the risk of future incident AF. This scoping review synthesizes evidence from original studies evaluating AI models trained on sinus rhythm ECGs for AF detection or AF prediction. *Materials and Methods:* A comprehensive search of MEDLINE, Embase, Web of Science, Scopus, and IEEE Xplore was conducted to identify peer-reviewed studies from inception to November 2025. Eligible studies included original investigations in which the model input was a sinus rhythm ECG and the outcome was either paroxysmal AF or new-onset AF. Extracted variables included cohort characteristics, ECG acquisition parameters, AI architecture, model predictive performance, AF prediction horizon, clinical outcomes, and validation strategy. Risk of bias was assessed using PROBAST. *Results:* Nineteen studies met the inclusion criteria. Retrospective datasets ranging from several thousand to over one million ECGs and convolutional or deep neural network AI architectures were used in most studies. AI-ECG models demonstrated high diagnostic accuracy for detecting subclinical AF (ten studies; AUROC 0.75–0.90) and for predicting long-term new-onset AF (six studies; AUROC 0.69–0.85) from a single sinus rhythm ECG. Robust external validation was reported in eleven studies. Combining AI-ECG models with clinical risk factors improved AF predictive performance in several reports. Key limitations across studies included retrospective design, patient selection, limited calibration reporting, and sparse prospective impact data. *Conclusions:* AI-based analysis of sinus rhythm ECGs can detect occult AF and stratify future AF risk with moderate-to-high accuracy across multiple populations and healthcare systems. However, rigorous prospective trials, evaluating clinical benefit, cost-effectiveness, calibration across demographic groups, and real-world implementation, are required before broad adoption in clinical practice.

## 1. Introduction

Atrial fibrillation (AF) is the most common sustained cardiac arrhythmia and a mounting global public-health challenge [1]. The lifetime risk of AF is approximately 1 in 3–5 individuals after the age of 45 years, and the prevalence is projected to rise substantially with increasing age and the burden of comorbidities [2]. AF is associated with a nearly five-fold increase in risk of ischaemic stroke [3,4] and a twofold increase in all-cause mortality [2,5]. Furthermore, increased risk of heart failure, reduced quality of life, and increased risk of cognitive impairment have been reported in patients with AF [1,5]. Up to 15–30% of patients with AF are undiagnosed until complications emerge [6,7], which highlights the inadequacy of current screening strategies.

Electrocardiogram (ECG), a low-cost, rapid and simple test, remains a cornerstone in cardiac arrhythmia diagnosis [1]. However, the subtle atrial substrate changes preceding AF may be overlooked during interpretation of sinus rhythm ECGs. Experimental and clinical studies have shown that atrial fibrosis and electrical remodelling lead to prolonged intra-atrial conduction, nonuniform anisotropy, and heterogeneity of depolarization, which manifest as changes in P-wave morphology and PR-interval dynamics [8,9].

Artificial intelligence (AI), especially deep learning methods, has revolutionized the analysis of high-dimensional physiological signals, enabling detection of latent disease phenotypes invisible to the human eye [10]. AI-based ECG algorithms have demonstrated remarkable accuracy for the detection of acute ischemia, left ventricular dysfunction, electrolyte abnormalities, and cardiac arrhythmias [11]. Furthermore, AI-based models have been recently used to detect paroxysmal atrial fibrillation from input sinus rhythm ECGs. A seminal study from the Mayo Clinic applied a convolutional neural network (CNN) to over 600,000 12-lead sinus rhythm ECGs and identified patients with a history of AF with an area under the curve (AUC) of 0.87 [12]. This approach has been validated across different databases and patient populations and extended to the prediction of new-onset AF in subsequent studies [13,14,15,16,17]. The 2024 ESC Guidelines on the management of AF recognize AI-based ECG analysis as an emerging tool for precision screening and risk stratification in patients prone to developing AF [1].

Nevertheless, the translation of AI-enabled ECG screening into clinical practice remains challenging. Model performance depends on heterogeneity of training datasets, ECG signal acquisition, and the time horizon for AF prediction. Furthermore, interpretation of results from reported studies is complicated by differences in external validation, calibration, and reporting standards [10,18,19]. Prospective studies assessing whether AI-driven ECG screening leads to improved outcomes (e.g., stroke and heart failure prevention, all-cause mortality benefits) are lacking [19,20].

Therefore, a comprehensive synthesis of published evidence is essential to define the current state of the field, assess the methodological quality of reported studies, evaluate the performance of different AI models, identify key gaps in the existing literature, and inform prospective implementation studies.

## 2. Materials and Methods

This scoping review was conducted in accordance with the PRISMA 2020 guidelines and adhered to standards for reporting AI-based diagnostic and prognostic studies (TRIPOD-AI). The protocol was prospectively developed before data extraction and predefined the research question, eligibility criteria, search strategy, data extraction protocol, and risk of bias assessment. The review protocol was registered retrospectively on the Open Science Framework (registration DOI: 10.17605/OSF.IO/VUJWG) to enhance transparency.

### 2.1. Research Question

Among adults with normal sinus rhythm on the index ECG, how accurately do AI models detect subclinical AF or predict new-onset AF?

### 2.2. Eligibility Criteria

#### 2.2.1. Inclusion Criteria

- Population: Adults (≥18 years) with baseline ECG recorded in sinus rhythm (no sustained arrhythmia on the input tracing). Multiple ECGs per patient were allowed.
- Index test/intervention: Any AI or machine learning model (e.g., CNN, DNN…) trained on and applied to ECG data (raw signals or ECG images).
- Comparator: Not mandatory. When present, comparators could include clinical risk scores (e.g., CHARGE-AF), polygenic risk scores, or clinician interpretation.
- Outcomes:
  - Detection of paroxysmal AF and/or short-term AF risk prediction (subclinical/latent disease already present; AI-based models could either detect the presence of paroxysmal AF or the short-term onset of known paroxysmal AF (time of AF onset < 1 year from the baseline ECG recording));
  - Prediction of long-term new-onset AF (risk stratification for long-term future/new-onset disease; none of the included patients supposedly had AF during baseline ECG recording (time of AF onset > 1 year from the baseline ECG recording));
  - Clinical implementation outcomes when AI-ECG was applied prospectively.
- Study design: Retrospective or prospective cohort studies, diagnostic accuracy studies, and interventional/implementation trials.
- Publication: Full-length, peer-reviewed articles in English.
- Time frame: Inception to 16 November 2025.

#### 2.2.2. Exclusion Criteria

- Case reports, narrative reviews, editorials, conference abstracts.
- Studies including datasets with AF (or other sustained arrhythmia) on the input ECG.
- Studies not using a standard 12-lead ECG for input tracing in the training cohort.
- Animal studies or purely simulated datasets.

### 2.3. Search Strategy

The search was performed in MEDLINE (via PubMed), Embase, Web of Science Core Collection, and IEEE Xplore from inception to 16 November 2025. We combined controlled vocabulary and free-text terms for AF, ECG, sinus rhythm, and AI, and adapted syntax to each database.

The core PubMed strategy was as follows:

(“atrial fibrillation”[MeSH Terms] OR “atrial fibrillation”[Title/Abstract]) AND (“electrocardiography”[MeSH Terms] OR “electrocardiogram”[Title/Abstract] OR “ECG”[Title/Abstract]) AND (“sinus rhythm”[Title/Abstract]) AND (“artificial intelligence”[MeSH Terms] OR “machine learning”[Title/Abstract] OR “deep learning”[Title/Abstract] OR “neural network”[Title/Abstract])

We added filters for humans, adults, English, and excluded obvious non-research article types. The search was supplemented by backward citation tracking of key primary studies and existing systematic reviews.

### 2.4. Study Selection

Two reviewers independently screened all titles and abstracts for relevance, followed by a full-text assessment against the eligibility criteria. Disagreements were resolved by a consensus.

### 2.5. Data Extraction

From each included study, two reviewers independently extracted the following:

- Study characteristics: year, country, setting, design, sample size, inclusion/exclusion criteria, follow-up.
- ECG data: duration, sampling frequency and filters, sinus rhythm and AF definition.
- AI model: architecture (CNN, DNN …), training/validation cohort splits, availability of explainability methods.
- Validation method: internal and/or external validation.
- Reference standard for AF: prior AF diagnosis, AF on other ECGs, AF on long-term monitoring or insertable cardiac monitors.
- AF prediction horizon (if applicable).
- Predictive performance: AUC (AUROC), sensitivity, specificity, calibration metrics.
- Clinical outcomes (if reported): AF-related stroke, mortality, cognitive decline, or AF detection yield in implementation trials.
- Funding sources and potential conflicts of interest.

### 2.6. Risk of Bias

Because the primary outcome is risk prediction/diagnostic performance, PROBAST (Prediction model Risk of Bias ASsessment Tool–Version of 15/05/2019) was used to broadly assess the risk of bias for each included study. Domains included participant selection, predictors (ECG and clinical variables), outcome definition, and analysis. Discrepancies were resolved by a consensus.

### 2.7. Data Synthesis and Analysis

Given the marked heterogeneity in datasets, AI architectures, study outcomes, and AF prediction and detection horizons, a quantitative meta-analysis was not planned. We prespecified a primarily narrative synthesis. Three major groups of outcomes were identified (AF detection, AF prediction, and clinical implementation outcomes). The heterogeneity of included studies was assessed qualitatively by visual inspection of forest plots.

## 3. Results

### 3.1. Study Selection

**Figure 1:**
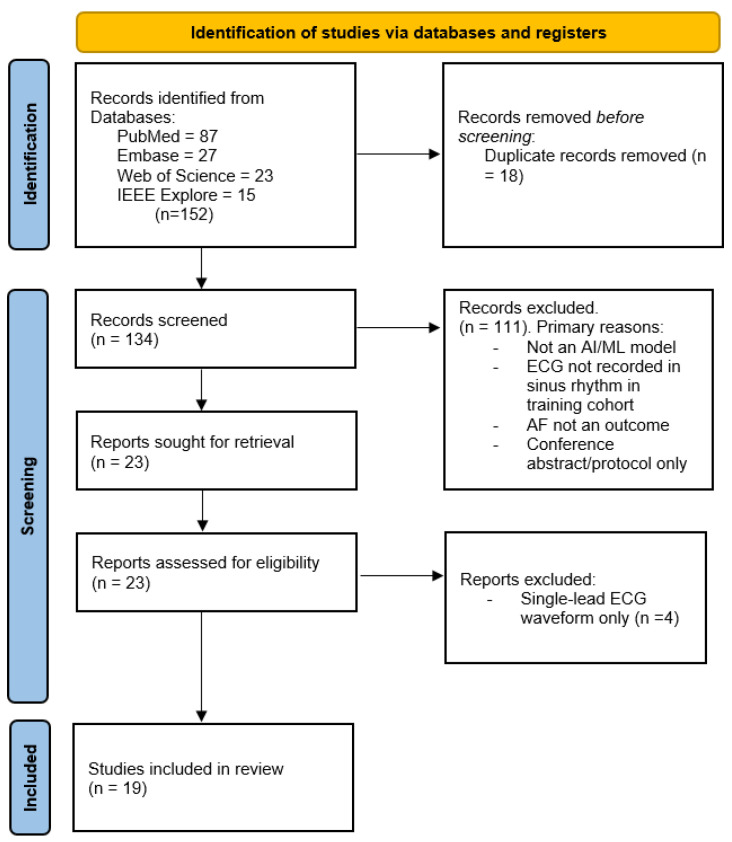
PRISMA Flow diagram. PRISMA—preferred reporting items for systematic reviews and meta-analyses; AI—artificial intelligence; ML—machine learning; ECG—electrocardiogram; AF—atrial fibrillation.

The database search and citation tracking identified 154 potentially relevant articles. After removal of duplicates and two-stage screening (title/abstract and full text), 19 studies met all predefined eligibility criteria (Table S1). These studies evaluated AI models applied to ECGs recorded during sinus rhythm to detect subclinical AF or to predict incident AF across a wide range of healthcare settings. The screening process is summarized by the PRISMA flow diagram (Figure 1).

### 3.2. Study Characteristics

#### 3.2.1. Settings and Patient Populations of the Included Studies

Across the 19 studies, patient populations varied widely. Training and validation cohorts included datasets from community-based cohorts (Mayo Clinic Study of Aging [21,22], UK Biobank [16] …), large health-system datasets (Mayo Clinic [12], Mass General Brigham [14] …), multinational hospital cohorts [23], and high-risk clinical subsets [23,24]. Sample sizes ranged from a few thousand [25,26] to hundreds of thousands or millions of ECGs [12,13,27]. Mean patient age typically ranged from 60 to 75 years, and almost all studies included patients at increased baseline risk for AF.

#### 3.2.2. ECG Modalities

The vast majority of studies used 10 s, 12-lead resting sinus rhythm ECGs in both training and testing cohorts [12,13,14,15,16,17,25,26,27] Sinus rhythm 12-lead ECGs were used as a training cohort, and an AI-based model to detect AF from a single-lead ECG was developed in two studies [28,29]. All studies defined sinus rhythm at the time of ECG acquisition; no study included patients with AF in model input ECG.

#### 3.2.3. AI Model Types

AI models included the following:

- Convolutional neural networks (CNNs): the dominant architecture [12,13,15,23,27].
- Deep neural networks (DNNs): often end-to-end waveform models [14,16,27].
- Transformer AI [29].
- Combined ECG + clinical risk models [14,15,30].

#### 3.2.4. Outcomes and Validation

Three major categories of study outcomes were reported:

- Detection of subclinical paroxysmal and/or prediction of short-term AF risk from a single sinus rhythm ECG [12,26,27,28,31].
- Long-term new-onset AF prediction from a single sinus rhythm ECG [13,14,15,21].
- Clinical implementation outcomes (e.g., AF detection yield, stroke prevention, cognitive decline), when AI-ECG AF scores were used prospectively in clinical practice [17,22,29].

External validation was present in 11 out of 19 studies (Table S1). Large international databases were used as testing cohorts in several studies [16,23,27]. Therefore, this evidence base was suited for evaluating generalizability.

### 3.3. Performance of AI Models

The discriminative performance of AI-based models is summarized in Figure S1.

#### 3.3.1. Detection of Paroxysmal AF and/or Short-Term Future AF Risk Prediction

Ten studies evaluated AI models to detect occult and/or predict short-term AF risk based on a sinus rhythm ECG input.

- Attia et al., 2019 [12]: CNN achieved AUROC 0.87 for detecting AF from a single sinus rhythm ECG. Model performance improved to ≈0.90 when the ECG was recorded close in time (30 days) to a documented AF episode.
- Baek et al., 2021 [26]: DNN achieved AUROC 0.75–0.79 for identifying paroxysmal AF. The AI-based model focused on P-wave morphology.
- Gruwez et al., 2023 [31]: External validation of the Mayo AI-ECG-AF algorithm in Belgium showed AUROC 0.87 for detection of subclinical AF.
- Hygrell et al., 2023 [28]: CNN was trained on 12-lead ECGs and tested on a single ECG lead device. AI-based model achieved AUROC 0.80 for detecting paroxysmal AF in the age-diverse SAFER cohort but only 0.62 in the age-homogenous STROKESTOP cohorts.
- Aminorroaya et al. [23], 2025: CNN, trained on 12-lead ECG images, accurately detected AF/atrial flutter with AUROC 0.80.
- Jin et al., 2025 [32]: DNN achieved AUROC 0.90 to predict the onset of known paroxysmal AF within one month of a normal sinus rhythm ECG.
- Chang et al., 2025 [24]: CNN model achieved AUROC 0.86 to detect past episodes of AF and AUROC 0.85 to predict future AF events in patients with paroxysmal AF.
- Cai et al., 2020 [25]: DNN, trained on 12-lead ECG images, accurately predicted subclinical AF with AUROC ≈ 0.90 in a small cohort.
- Yuan et al., 2023 [27]: CNN accurately detected paroxysmal AF and predicted its onset within one month, achieving AUROC 0.86, with multiple external validations ranging 0.83–0.93.
- Tarabanis et al., 2025 [30]: CNN + CHARGE-AF clinical score improved paroxysmal AF detection (AUROC 0.89) relative to either ECG-AI (AUROC 0.83) or CHARGE-AF alone.

Across different settings and patient cohorts, AI models consistently detected subclinical AF from sinus rhythm ECGs with AUROC ≈ 0.75–0.90. Cross-system generalizability was demonstrated with robust external validation in several studies.

#### 3.3.2. Prediction of Long-Term New-Onset AF

Six studies focused on long-term new-onset AF prediction from a single sinus rhythm ECG.

- Christopoulos et al., 2020 [21]: CNN model predicted AF over 14-year follow-up with AUROC 0.69., measuring up to clinical CHARGE-AF score. Furthermore, combining the AI-based model with CHARGE-AF outperformed both modalities (AUROC 0.72).
- Raghunath et al., 2021 [13]: DNN predicted 1-year new-onset AF with AUROC 0.80–0.85. In all patients who experienced AF-related stroke, the model predicted high risk for new-onset AF in 62% patients.
- Khurshid et al., 2022 [14]: CNN alone achieved AUROC 0.71–0.82 and was comparable to CHARGE-AF. Combination of ECG-AI and clinical risk model improved prediction of new-onset AF (AUROC 0.75–0.84).
- Jabbour et al., 2024 [15]: ECG-AI alone yielded AUC 0.73–0.78; combining ECG, clinical variables, and polygenic score produced AUC ≈ 0.78 and improved model calibration.
- Brant et al., 2025 [16]: Multinational validation (FHS, UKBB, ELSA-Brasil) of a DNN ECG model showed AUROC 0.80–0.82 across heterogeneous populations. The model was complementary to CHARGE-AF (AUROC 0.85).
- Habineza et al., 2023 [33]: CNN-based model, trained and evaluated on a large cohort, achieved AUC 0.85 for predicting new-onset AF in seven years.

Across six high-quality studies with external validation, long-term new-onset AF prediction from a single SR ECG consistently achieved AUROC ≈ 0.69–0.85, often measuring up to established clinical risk scores. Furthermore, combining AI-based models with clinical risk scores additionally improved model performance.

#### 3.3.3. Clinical Implementation Studies

Three studies reported prospective clinical implementation of AI-ECG models:

- Noseworthy et al., 2022 [17]: AI-guided risk stratification tripled AF detection (10.6% vs. 3.6%) over a median follow-up of 9.9 months in high-risk patients in a pragmatic, real-world trial.
- Weil et al., 2022 [22]: High-risk AI-ECG AF scores predicted cognitive impairment and greater brain infarct burden, suggesting relevance to AF-related target organ damage.
- Choi et al., 2024 [29]: AI applied to sinus rhythm ECG in patients with embolic stroke of unknown source (ESUS) improved selection for prolonged monitoring, achieving high occult AF yield.

A limited number of studies discussed the clinical utility of AI-based models in selective AF screening. However, reports evaluating hard clinical outcomes (e.g., mortality and stroke reduction) and prospective trials are lacking.

### 3.4. Risk of Bias and Applicability

Across the 19 included studies, risk of bias was broadly assessed using the PROBAST tool (Table S2).

- Participant selection: Studies usually included retrospective datasets from high-income countries; data from low-resource countries is limited. The majority of studies focused on patients who were at high risk for new-onset AF or those with subclinical AF.
- Predictors: AI-based models were often trained on large cohorts. However, using multiple ECGs per patient was variably reported.
- Outcomes: Sinus rhythm and AF were verified in all studies. AF detection and prediction were assessed across different patient populations. However, several studies included a limited number of ECG records.
- Analysis: Internal validation was performed in most studies; external validation was reported in 11 studies. Calibration and decision-curve reporting were inconsistent. The number of participants with the outcome was limited in several studies due to a small testing cohort and a low prevalence of paroxysmal AF.

Overall, risk of bias was low in four studies, high in eight studies, and unclear in seven studies. Concerns regarding applicability were present in 2/16 studies. Although the majority of studies were applicable to the review question, major risk of bias was possible in over 3/4 of the included reports.

## 4. Discussion

This scoping review synthesized evidence from studies that applied AI algorithms to standard ECGs recorded during sinus rhythm to either detect subclinical AF or predict new-onset AF. Across the 19 studies included in this review, the principal finding is consistent. AI-ECG models trained on large datasets achieve good discrimination for identifying patients with occult/paroxysmal AF (ten studies; AUROC ≈ 0.75–0.90) or future/new-onset AF (six studies; AUROC ≈ 0.69–0.85). The findings of Attia and colleagues [12], who first showed that a CNN could detect paroxysmal AF from sinus rhythm ECGs with high diagnostic accuracy, have been corroborated in multiple diverse independent cohorts across North America, Europe, and Asia and extended to the prediction of new-onset AF [13,14,15,16,21,23,24,25,26,27,28,30,31,32,33]. Furthermore, three studies discussed the clinical utility of AI-based models in selective screening [17,22,29].

### 4.1. Model Explainability and ECG Substrate

AF detection and prediction from sinus rhythm ECGs are based on the concept that atrial structural and electrical remodelling alter surface ECG morphology before arrhythmia onset. Decreased atrial conduction velocity, shortening of the atrial effective refractory period, and increased dispersion of refractoriness can be explained by atrial fibrosis, myocyte hypertrophy, and anisotropic conduction [34,35]. These abnormalities are reflected on the 12-lead ECG as a subtle prolongation and fragmentation of the P-wave, altered inter-atrial conduction patterns, and variability in PR intervals [36,37]. In population-based cohorts, such as Framingham and Copenhagen ECG studies, prolonged P-wave duration and PR-interval length were independently associated with new-onset AF [8,9,38].

AI-enabled ECG algorithms have been traditionally viewed as ˝black box˝ models, offering little insight into which ECG waveform characteristics drive predictions. Without explainability, clinicians may struggle to trust AI-derived predictions, which limits widespread adoption in clinical practice. Therefore, several recent studies calculated saliency maps to enhance the model transparency and identify the area on the ECG which had the largest impact on outcome prediction [14,15,24,31]. Saliency mapping revealed that the terminal part of the P-wave area, which corresponds to the depolarization of the left atrium, had the greatest effect on paroxysmal AF detection in the external validation of Mayo Clinic CNN [31]. Additionally, P-wave duration and amplitude in leads II and V1 were found to be associated with AF detection. Furthermore, saliency maps demonstrated that the P-wave area and the surrounding regions had the highest influence on AI-based new-onset AF prediction [14,15]. Individuals with high estimated AF risk tended to have a longer P-wave duration, larger P-wave amplitude, as well as slightly wider QRS and a flatter ST segment. Moreover, the layer-wise relevance propagation (LRP) method was recently used for enhancing interpretability of an AI-based algorithm designed for paroxysmal AF detection [32]. The LRP is a prominent method for interpreting deep learning models by measuring the contribution of each input to the output of the model. The T-wave area, as well as the ST segment and the P-wave area, were shown to have the greatest influence on outcome prediction. Finally, it was reported that a higher risk for paroxysmal AF, as assessed by an AI-based model, was associated with greater atrial myopathy on echocardiography and invasive testing [39]. These findings support the hypothesis that AI networks might exploit physiologic markers of atrial disease. However, prospective clinical trials using large international datasets and explainable AI models are warranted to truly explain model outcome prediction and establish user trust in AI-based diagnostic systems.

### 4.2. Model Discrimination and Comparison with Clinical Risk Scores

Across the included studies, model discrimination was generally high. An AUC of 0.87 for AF detection from sinus rhythm ECGs using a CNN was reported by the Mayo Clinic group [12]. Aforementioned machine learning model was externally validated in a subsequent report, achieving similar predictive accuracy (AUROC 0.87) [31]. Paroxysmal AF detection from a single sinus rhythm ECG using a newly developed CNN was the outcome of another study [24]. The AI-based algorithm, trained on nearly 1 M ECGs, achieved an AUROC 0.86 for detecting past AF episodes and an AUROC 0.85 for predicting 1-year future AF risk in patients with known AF. Recently, a CNN was developed to detect paroxysmal AF from sinus rhythm ECGs and predict the risk of a future AF episode, occurring within 1 month [31]. The algorithm demonstrated high accuracy (AUROC 0.90).

Long-term new-onset AF prediction from a single sinus rhythm ECG using the modified Mayo Clinic CNN was first reported in 2020 (AUROC 0.69) [21]. In a subsequent study using 1.6 M ECGs from the MUSE database, a DNN was developed to predict new-onset AF (within 1 year) with an AUROC of 0.80–0.83 [13]. In this population, it outperformed the clinical risk score CHARGE-AF. A CNN ECG-AI model was tested against the CHARGE-AF clinical model to infer 5-year incident AF in another report [14]. AI-based analysis of the 12-lead ECG had a similar predictive usefulness to the clinical risk factor model. However, the highest AUROC (0.84) was achieved by combining both modalities, suggesting both approaches were complementary.

Subsequent multicentre evaluations demonstrated consistent performance of different AI models to either predict new-onset AF or detect paroxysmal AF across different populations and ECG datasets [16,23,27,30]. In contrast, smaller single-centre datasets or models with limited training datasets and diversity showed reduced performance (AUROC 0.75–0.80) [25,26]. AI-based models were compared with the standard clinical risk score CHARGE-AF in a handful of studies [13,14,15,16,30]. Although rarely reported, the integration of CHARGE-AF components into the CNN model was associated with a greater predictive accuracy for incident AF [15,40].

### 4.3. Generalization and Dataset Heterogeneity

A key challenge is ensuring model robustness across healthcare systems and devices. Deep learning algorithms trained and tested on one dataset often perform worse when applied to external data, owing to differences in ECG acquisition parameters, demographic distribution, and comorbidity prevalence [41]. AI-based algorithms trained on non-representative datasets may struggle to perform reliably in real-world populations, leading to misdiagnosis through false positives or negatives. Superior generalizability with AUC maintained above 0.80 across datasets was achieved in studies that enabled model training with a large database from different institutions [13,16,30]. Imbalanced datasets with relatively rare AF events may also negatively impact model robustness and performance [40]. In studies evaluating paroxysmal AF detection, an AF prevalence of approximately 10% was reported in the testing cohort [12,31]. The global AF burden is estimated at 1–2% [2]. When testing the algorithm in a population with an AF prevalence of 3%, the algorithm performance deteriorated and the area under the precision–recall curve (AUPRC) decreased [31]. Therefore, AI-based algorithms might be best suited for AF detection in groups with similar AF prevalence to the training cohort, unless model recalibration is performed.

Furthermore, demographic characteristics of the training cohort influence model generalizability. Variations in age, sex, ethnicity, and underlying comorbidities can influence both the electrophysiological characteristics of cardiac conduction captured by the ECG, such as P-wave duration, P-wave amplitude, and PR interval, and the AF prevalence [8,9,36]. Demographic analyses were performed in several trials [12,13,14,21]. Slightly higher sensitivity in younger patients and consistent performance between males and females using a CNN for paroxysmal AF detection were noted by the Mayo Clinic group [12]. The overall accuracy of a new-onset AF prediction model was maintained across different ethnic subgroups in another study [21]. However, minor differences in specificity were reported. A DNN algorithm retained its ability to discriminate between high- and low-risk populations for future AF across different age groups in both men and women [13].

Moreover, standardizing input features or developing benchmark datasets would support model generalizability across diverse patient populations. Finally, integration of clinical and laboratory parameters into multimodal models may improve model discrimination, generalization and interpretability, as pointed out in a recent study employing a small dataset [40]. However, temporal external validation, using new data collected years after model training, was seldom reported [21,31], which limits the generalizability of reported models.

### 4.4. Model Calibration

The process of adjusting model parameters to better align model outputs with the true likelihood of an event represents an important part in building reliable, robust AI-based algorithms. Across the included studies, model calibration was inconsistently reported. Only a minority of studies included calibration slope statistics (calibration curves) [13,14,27,30]. Therefore, some models trained on high-risk groups may overestimate absolute AF probability in low-risk groups, suggesting a need for recalibration before clinical deployment [12,14,15]. A substantial overestimation of a new-onset AF risk in a low-risk testing sample was demonstrated in a report [14]. However, simple recalibration to the baseline AF hazard in the low-risk cohort substantially improved calibration and the integrated calibration index. Interestingly, the CHARGE-AF clinical risk score had worse calibration even after recalibration in the low-risk sample, suggesting that AI-based AF risk may result in more calibrated estimates [14]. In another report, evaluating new-onset AF prediction, the addition of a clinical risk score to the AI-enabled ECG model improved the calibration performance and reduced the estimated calibration index [15]. Across different testing cohorts, the calibration performance of a deep learning algorithm, used for paroxysmal AF detection, was assessed using a Brier score (Spiegelhalter z test) [27]. Strong calibration performance, with predicted probabilities closely matching paroxysmal AF prevalence across various datasets, was recently reported in a CNN used for AF detection [30].

Nevertheless, the change in data distribution between the training cohort and the testing cohort, also known as a cross-population domain shift, was present in some of the studies [14,28,30]. An AI-based algorithm, which was trained on a limited number of ECGs from the age-homogenous STROKESTOP II group, demonstrated modest accuracy (AUROC = 0.62) for paroxysmal AF detection in both STROKESTOP I and STROKESTOP II cohorts [28]. These gaps underscore the need for standardized model reporting as outlined in the TRIPOD-AI statement [42].

### 4.5. AI-ECG Screening

AI-ECG screening may complement conventional AF screening strategies and help to identify individuals at high risk before arrhythmia onset or detect those with paroxysmal subclinical AF. It was recently reported in a small nonrandomized interventional study that patients with stroke risk factors flagged by a CNN AI-ECG model were over four times more likely to develop AF during a mean of 22 days of continued monitoring compared with those classified as low-risk [17]. Furthermore, AI-guided screening was associated with increased detection of subclinical AF compared with usual care over a median follow-up of 9 months. In another study, an AI-enabled ECG model predicted high risk for underlying paroxysmal AF in the majority of patients with embolic stroke of unknown source (ESUS), who were later diagnosed with AF on an implantable cardiac monitor [29]. Furthermore, the algorithm was used to predict the future new-onset development of AF within a 2-year period and risk-stratify the patients. Interestingly, the AI-based AF risk increased as the ECG recording approached the AF onset. Continuous surveillance and screening of high-risk populations could be achieved by combining AI-enabled ECG models with commercially available single-lead monitors (e.g., smartwatches) [43]. AI-based ECG models, trained on 12-lead ECGs and used to detect AF on a single-lead ECG, achieved an AUC 0.75–0.8 in two reports [28,29].

### 4.6. Limitations of Current Evidence

First, retrospective ECG datasets were used in most of the published studies and patients with structural heart disease were not always included (selection bias). Second, pre-specified external validation and prospective follow-up were seldom reported. Therefore, the accuracy of models to predict new-onset AF or detect subclinical AF may have been overestimated in some cases. Third, studies comparing one AI-based ECG model to another are lacking. Fourth, calibration plots, cost-effectiveness analyses, and assessment of clinical impact were not always reported, limiting the real-world implementation of the presented models. Fifth, key performance metrics, such as AUPRC, PPV, and NPV, were seldom reported. In highly imbalanced (positive ≪ negative cases) training and testing datasets, algorithms may achieve misleadingly high AUROC, but not a high AUPRC. Sixth, several studies used ECGs recorded very close to AF diagnosis (within 30 days) and included multiple ECGs per patient without addressing intra-subject dependency (temporal bias). Therefore, data leakage might have been introduced, which can cause inflated performance estimates. Seventh, robust randomized trials demonstrating clinical benefit are lacking. Accordingly, current evidence is hypothesis-generating and indirect, rather than outcome-proven.

### 4.7. Future Directions

As emphasized by the 2024 ESC AF guidelines [1], AI-based ECG models represent a promising tool for arrhythmia prevention and risk stratification but require rigorous validation before clinical endorsement. Future research should focus on large, prospective, randomized, multicentre studies integrating AI-ECG prediction into clinical workflows. Prospective studies employing AI-based models with large heterogeneous training datasets could improve model discrimination and generalizability across various demographic groups. Combining ECG-based AI with imaging and biochemical biomarkers and genetic data could yield integrated risk models with superior predictive power. Nonetheless, existing AI-based models should be tested across diverse populations to prove the reproducibility of reported results.

Ongoing trials such as DEEP-AF (NCT07173673) are expected to clarify whether early identification of at-risk patients improves subsequent AF detection. The utility of long-term monitoring using AI-enabled algorithms and smartwatches to detect AF is investigated in another study (REGAL, NCT05923359). Predicting AF progression to personalize patient management using AI-based ECG and imaging data is the focus of another ongoing trial (NCT06647914).

## 5. Conclusions

AI applied to sinus rhythm ECGs offers a powerful and non-invasive tool of identifying paroxysmal AF and predicting long-term new-onset AF. This scoping review based on prospective, non-randomized, single-centre studies supports the strong discriminative performance of AI-based ECG models. However, prospective randomized trials are lacking, and the current evidence should be viewed as merely hypothesis-generating. Future clinical trials will determine whether AI-ECG models can become a part of everyday clinical care.

## Data Availability

No new data were created or analyzed in this study. Data sharing is not applicable to this article.

## Supplementary Materials

The following supporting information can be downloaded at https://www.mdpi.com/article/10.3390/medicina62010199/s1: Table S1—Included studies: ECG—electrocardiogram; AI—artificial intelligence; AF—atrial fibrillation; SR—sinus rhythm; CNN—convolutional neural network; AUROC—area under a receiver operating characteristic curve; DNN—deep neural network; AI-ECG—artificial intelligence-enabled electrocardiogram; ICM—implantable cardiac monitor. Table S2—PROBAST-based risk of bias assessment and applicability concerns: PROBAST—Prediction model Risk of Bias Assessment Tool. Figure S1—Forest plots of outcome measures for all included studies: AUROC—area under a receiver operating characteristic curve; CI—confidence interval.
