# Supplementary material for: Artificial Intelligence Applied to Electrocardiograms Recorded in Sinus Rhythm for Detection and Prediction of Atrial Fibrillation: A Scoping Review
Source: Medicina (Kaunas). 2026 Jan 17;62(1):199. doi: 10.3390/medicina62010199 (PMC12843910; doi:10.3390/medicina62010199)
Supplement: Supplementary file 1 [file medicina-62-00199-s001.zip › medicina-4093003-supplementary.pdf]

TABLE S1 - Included studies

Study type	Study (Year)	Input ECG model	Population / Setting	AI Model Type	Target Outcome	AF Prediction Time Horizon	Validation	Performance Summary
Paroxysmal AF detection and/or short-term future AF risk	Attia et al., (2019) [12]	12-lead ECG, SR	U.S. health system (Mayo Clinic)	CNN	Detection of paroxysmal AF	Not applicable	Internal + External	AUROC 0.87
	Cai et al., (2020) [25]	12-lead ECG images, SR	Small cohort	DNN	Detection of paroxysmal AF	Not applicable	Internal	AUROC ≈ 0.90
	Baek et al., (2021) [26]	12-lead ECG, SR	Korean hospital cohort	DNN	Detection of paroxysmal AF	Not applicable	Internal	AUROC 0.75–0.79
	Gruwez et al., (2023) [31]	12-lead ECG, SR	Belgian external validation of Mayo AI-ECG	CNN	Detection of paroxysmal AF	Not applicable	External	AUROC 0.87
	Yuan et al., (2023) [27]	12-lead ECG, SR	U.S. Veterans	DNN	Detection of paroxysmal AF + future 1-month AF risk	31 days (future AF risk)	Internal + External	AUROC 0.86 (external 0.83–0.93)
	Hygrell et al., (2023) [28]	12-lead ECG, SR – training cohort; single-lead ECG – testing cohort	SAFER & STROKESTOP screening cohorts	CNN	Detection of paroxysmal AF	Not applicable	External	AUROC 0.62–0.80
	Aminorroaya et al., (2025) [23]	12-lead ECG, SR	Multinational cohorts	CNN	Detection of AF/atrial flutter	Not applicable	Internal + Multinational external	AUROC 0.80

Study type	Study (Year)	Input ECG model	Population / Setting	AI Model Type	Target Outcome	AF Prediction Time Horizon	Validation	Performance Summary
	Jin et al., (2025) [32]	12-lead ECG, SR	AF clinical cohort	DNN	Detection of past AF episodes + future 1-month AF risk in patients with paroxysmal AF	31-days (future AF risk)	Internal	AUROC 0.90
	Tarabanis et al., (2025) [30]	12-lead ECG, SR	European multi-hospital datasets	CNN+ clinical	Detection of paroxysmal AF	Not applicable	Internal + External	AUROC 0.83
	Chang et al., (2025) [24]	12-lead ECG, SR	Multicenter dataset	CNN	Detection of past AF episodes + future AF risk in patients with paroxysmal AF	1 year (future AF risk)	Internal + External	AUROC 0.86 (past AF); 0.85 (1-year future AF risk)
Long-term new-onset AF prediction	Christopoulos et al., (2020) [21]	12-lead ECG, SR	Mayo Clinic Study of Aging	CNN	Incident/future AF risk	14 years	Internal	AUROC 0.69
	Raghunath et al., (2021) [13]	12-lead ECG, SR	Geisinger Health System (MUSE database)	DNN	Incident/future AF risk	1 year	Internal + External (stroke analysis)	AUROC 0.80–0.85
	Khurshid et al., (2022) [14]	12-lead ECG, SR	Mass General Brigham, UK Biobank	CNN	Incident/future AF risk	Mean follow-up ~ 5 years	Internal + External	AUROC 0.71-0.82
	Habineza et al., (2023) [33]	12-lead ECG, non-AF rhythm	Brazil national CODE dataset	DNN	Incident/future AF risk	Up to 7 years	Internal	AUROC 0.85

Study type	Study (Year)	Input ECG model	Population / Setting	AI Model Type	Target Outcome	AF Prediction Time Horizon	Validation	Performance Summary
	Jabbour et al., (2024) [15]	12-lead ECG, SR	Montreal Heart Institute	DNN + clinical + genomic	Incident/future AF risk	5 years	Internal + External	AUROC 0.73-0.78
	Brant et al., (2025) [16]	12-lead ECG, SR	FHS, UK Biobank, ELSA	DNN	Incident/future AF risk	Median follow-up 5–10 years, varies by cohort	Internal + Multinational External	AUROC 0.80–0.82
Clinical implementation trials	Noseworthy et al., (2022) [17]	12-lead ECG, SR	U.S. Primary Care	CNN	AF detection yield	Not applicable	/	High-risk group AF detection 10.6% (AI-guided screening) vs 3.6% (standard care)
	Weil et al., (2022) [22]	12-lead ECG, SR	Mayo Clinic Study of Aging	CNN	Cognitive decline	Long-term tracking 7–10 yrs	/	High AI-ECG risk associated with cognitive decline & infarct size
	Choi et al., (2024) [29]	12-lead ECG, SR – training cohort; single-lead ECG (ICM) – testing cohort	U.S. Health system (ESUS patients)	Transformer AI	AF detection yield (current paroxysmal AF, future AF risk)	2 years	/	AI-guided risk stratification for prolonged monitoring in ESUS patients.

TABLE S2 - PROBAST-based Risk-of-Bias Assessment and Applicability concerns

Study (year of publication)	Risk-of-bias assessment					Applicability concerns		
	Population	Predictors	Outcome	Analysis	Overall	Population	Predictors	Outcome
Attia et al., (2019) [12]	Low	Low	Low	Low	Low	Low	Low	Low
Cai et al., (2020) [25]	Low	Low	Unclear	High	High	Unclear	Low	Low
Christopoulos et al., (2020) [21]	Low	Unclear	Low	Unclear	Unclear	Low	Low	Low
Baek et al., (2021) [26]	Low	Unclear	Low	High	High	Low	Low	Low
Raghunath et al., (2021) [13]	Low	High	Unclear	Low	High	Low	Low	Low
Khurshid et al., (2022) [14]	Low	Low	Low	Low	Low	Low	Low	Low
Noseworthy et al., (2022) [17]	Low	Low	Unclear	Low	Unclear	NA	NA	NA
Weil et al., (2022) [22]	Low	Unclear	Low	High	High	NA	NA	NA
Yuan et al., (2023) [27]	Unclear	Low	Low	Low	Unclear	Low	Low	Low
Hygrell et al., (2023) [28]	Low	Low	Unclear	Unclear	Unclear	Low	Low	Low
Gruwez et al., (2023) [31]	Low	Unclear	Low	Unclear	Unclear	Low	Low	Low
Habineza et al., (2023) [33]	Unclear	Low	High	Low	High	Low	Low	Low
Jabbour et al., (2024) [15]	Low	Low	Low	Low	Low	Low	Low	Low
Choi et al., (2024) [29]	Low	Low	Low	Unclear	Unclear	NA	NA	NA

Study (year of publication)	Risk-of-bias assessment					Applicability concerns		
	Population	Predictors	Outcome	Analysis	Overall	Population	Predictors	Outcome
Aminorroaya et al., (2025) [23]	Unclear	Low	High	Unclear	High	Low	Low	Unclear
Brant et al., (2025) [16]	Low	Low	Low	Low	Low	Low	Low	Low
Chang et al., (2025) [24]	Low	High	Unclear	Low	High	Low	Low	Low
Tarabanis et al., (2025) [30]	Low	Low	Unclear	Low	Unclear	Low	Low	Low
Jin et al., (2025) [32]	Unclear	Low	Low	High	High	Low	Low	Low

Figure S1 – Forest Plots of outcome measures for all included models

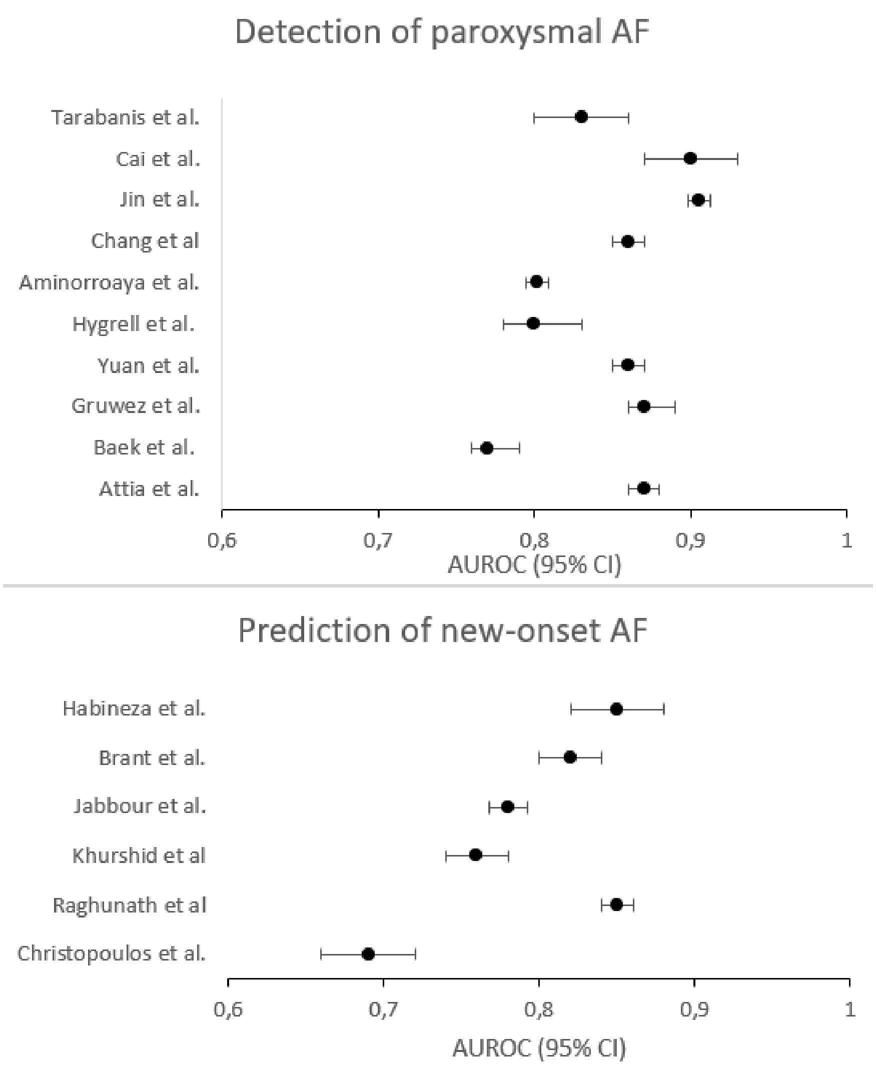
Detection of paroxysmal AF
Tarabanis et al.
Cai et al.
Jin et al.
Chang et al
Aminorroaya et al.
Hygrell et al.
Yuan et al.
Gruwez et al.
Baek et al.
Attia et al.
0,6
0,7
0,8
0,9
1
AUROC (95% CI)
Prediction of new-onset AF
Habineza et al.
Brant et al.
Jabbour et al.
Khurshid et al
Raghunath et al
Christopoulos et al.
0,6
0,7
0,8
0,9
1
AUROC (95% CI)
